# Silver nanoparticles in orthodontics, a new alternative in bacterial inhibition: in vitro study

**DOI:** 10.1186/s40510-020-00324-6

**Published:** 2020-08-17

**Authors:** Irania Jasso-Ruiz, Ulises Velazquez-Enriquez, Rogelio José Scougall-Vilchis, Raúl Alberto Morales-Luckie, Toshiko Sawada, Ryozo Yamaguchi

**Affiliations:** 1 Department of Health Sciences, School of Nursing, Autonomous University State of Mexico, Paseo Tollocan S/N, esq. Jesús Carranza, Moderna de la Cruz, 50180 Toluca City, México; 2 Department of Orthodontics, School of Dentistry, Autonomous University State of Mexico, Paseo Tollocan esq. Jesús Carranza S/N, Universidad, 50130 Toluca City, México; 3 Department of Nanomaterials, Sustainable Chemistry Research Center/National Autonomous University of Mexico, Highway Km. 14.5, Unidad San Cayetano, Toluca - Atlacomulco, 50200 Toluca City, México; 4 Department of Prosthodontics, School of Dentistry, Asahi University, 1851 Hozumi, Gifu, 501-0296 Japan; 5 Research Institute of Radioisotope, School of Dentistry, Asahi University, 1851 Hozumi, Gifu, 501-0296 Japan

**Keywords:** Orthodontic brackets, *Streptococcus mutans*, *Streptococcus sobrinus*, Silver nanoparticles, White spot lesion

## Abstract

**Background:**

The purpose of the study is to assess the antiadherent and antibacterial properties of surface-modified different orthodontic brackets with silver nanoparticles against *Streptococcus mutans* and *Streptococcus sobrinus*, using radiomarker.

**Methods:**

In this study evaluated quantitatively the adherence of *Streptococci* to orthodontic brackets, 300 samples of orthodontic brackets were selected and classified in to 10 groups as follow: GIn (InVu-Roth), GIIn (System-AlexanderLTS), GIIIn (Gemini-Roth), GIVn (NuEdge-Roth), GVn (Radiance plus-Roth), GVI (InVu-Roth), GVII (System-AlexanderLTS), GVIII (Gemini-Roth), GIX (NuEdge-Roth), GX (Radiance plus-Roth). All the samples were sonicated and *Streptococci* were cultivated by gender. A radioactive marker (^3^H) was used to codify the bacteria and measure them. After that, the brackets were submerged in a radiolabelled solution, and the radiation was measured. The statistical analysis was calculated with ANOVA test (Sheffè post hoc).

**Results:**

The results showed significant differences were found among the groups. GIIIn shown the lowest scores for both bacteria; in contrast, GIX for *Streptococcus mutans* and GVI for *Streptococcus sobrinus* were the highest values.

**Conclusions:**

Surface modification of orthodontic brackets with silver nanoparticles can be used to prevent the accumulation of dental plaque and the development of dental caries during orthodontic treatment.

## Background

The oral cavity environment provides certain essential characteristics for the proliferation of bacteria that are capable of producing acids that demineralize the surface of the tooth enamel [1]. Biofilm has a crucial role in the adhesion of these microorganisms to the dental surface [2, 3]. Enamel demineralization is caused by the organic acids produced by various microorganisms, mainly *Streptococcus mutans* (*S. mutans*) and *Streptococcus sobrinus* (*S. sobrinus*), which are identified as the main pathogens in dental caries [4–7].

Dental caries has been defined by the World Health Organization (WHO) as a localized process of multifactorial origin; this begins as a demineralization, which is the softening of the hard tissue of the tooth and evolves into the formation of a cavity. WHO reports that there is a prevalence in 60% to 90% of school children and almost 100% of adults have dental caries around the world, coinciding with the Official Mexican Standard 013 report, where it is mentioned that there is a 90% prevalence in Mexico [8].

Orthodontic treatment, using fixed appliances (brackets, bands, archwires, ties), provides suitable conditions to bring about the colonization of cariogenic microorganisms. Because fixed appliances promote the retention and adhesion of biofilm, dental hygiene becomes more complicated, and microorganisms increase the risk of enamel demineralization [4, 5].

Since 1985, the scientific community has been very concerned about the interaction between orthodontic devices and oral bacteria [9, 10]. In 2012, Freitas et al. concluded moderate evidence that the presence of fixed appliances influences the quantity and quality of oral microbiota [11]. Moreover, Luchese et al., in their research, report that orthodontic appliances influence the oral microbiota with an increase in the counts of *S. mutans* and *Lactobacillus spp*. and in the percentage of potentially pathogenic gram-negative bacteria [12].

It has been claimed that 50 to 70% of patients undergoing fixed orthodontic appliance therapy had enamel demineralization around the brackets (white spot lesions or cavities) [4, 5]. This has been widely known from the first month after the brackets placement, ranging from 12.6 to 50% [13–17].

The oral pH levels and various microorganisms normally present in the oral cavity may influence the adhesion capacity of bacteria, a formation of biofilm, which increases the risk of demineralization in enamel, and caries development, particularly the bracket material [18].

Electrostatic and hydrophobic interactions mostly cause the first affinity of bacteria to solid surfaces. Surfaces with high free energy attract bacteria, such as *S. mutans*, more easily [18]. In a study by Eliades et al. [19], stainless steel presented the highest critical surface tension and can be expected to have a higher plaque-retaining capacity. Metallic orthodontic brackets have been found to induce specific changes in the oral environment, such as reduced levels of pH, increased plaque accumulation, and elevated *S. mutans* and *S. sobrinus* colonization. Nevertheless, recent studies on possible differences in the initial affinity and adherence of bacteria on metal, ceramic, and plastic brackets over time were inconclusive [1, 19, 20].

The prevention of white spot lesions, caries, and periodontal problems during orthodontic treatment is a significant challenge to the clinician and the patient. Many strategies have been proposed and developed to minimize these biological consequences, which may include fluoride varnishes or mousses, various toothpastes, and mouth rinses [19, 21]. Unfortunately, only less than 15% of orthodontic patients follow instructions [22–24].

Besides that with the emergence of an antibiotic-resistant strain of bacteria, certain metals particularly in nanoparticle form have attracted attention. Nanoparticles are insoluble particles having a size smaller than 100 nm and can be used either combining with dental materials or by coating the surface which aims to reduce the microbial adhesion and prevent caries [14].

Among the various metals, silver since ages is known for its antimicrobial activity against gram-positive and negative bacteria, fungi, protozoa, and certain viruses, including antibiotic-resistant strains. Because of these properties, silver is widely used in burned areas, medical devices, textile fabric, and as a water purifier [25]. Surface coating of silver can be obtained by different methods, chemistry, physical, and biological [26].

The silver nanoparticles (AgNPs) have been added to conventional orthodontic adhesives and appliances, the critical issue is that the physical and chemical properties should not be affected adversely, leading to the ideal clinical performance. Further, the antimicrobial and antiadhesive properties, as well as the safety of the new nanoadhesives, must be ensured over a clinically relevant time span [27, 28].

As it has been found in the scientific literature, the studies on the use of AgNPs is limited [28–32]. It is for this reason that the purpose of this investigation was to determine and compare the independent bacterial colonization of *S. mutans* and *S. sobrinus* in five different types of orthodontic bracket materials, as well as to verify the effectiveness of the incorporation of AgNPs in some of them.

## Methods

### Orthodontic brackets

A total of 300 commercial orthodontic brackets were used (*n* = 30 per group) and classified into 10 groups of orthodontic brackets (5 groups with silver nanoparticles and 5 groups without silver nanoparticles) of the different material as follows: GIn InVu Roth, (TP Orthodontics, LaPorte, Ind., USA), GIIn System Alexander LTS (AO. American Orthodontics, Wisconsin., EE. UU), GIIIn Gemini Roth (3 M Unitek, Monrovia, CA., USA), GIVn Nu-Edge Roth (TP Orthodontics, LaPorte, IN, USA), GVn Radiance plus Roth (AO. American Orthodontics, Wisconsin., EE. UU), GVI InVu Roth (TP Orthodontics, LaPorte, IN, USA), GVII System Alexander LTS (AO. American Orthodontics, Wisconsin., EE. UU), GVIII Gemini Roth (3 M Unitek, Monrovia, CA, USA), GIX Nu-Edge Roth (TP Orthodontics, LaPorte, IN, USA), GX Radiance plus Roth (AO. American Orthodontics, Wisconsin., EE. UU).

### Preparation of samples

There is a total of 300 orthodontic brackets (150 brackets for *S. mutans*, 150 brackets for *S. sobrinus*); all the samples were initially cleaned ultrasonically for a minute to eliminate impurities and were then dried. Only half of the samples from each group that does not have silver nanoparticles were sterilized with ethylene oxide gas, the other half of the samples have silver nanoparticles (AgNPs). To avoid contamination, the samples were stored in a humidity-free environment.

### Radiolabeled bacteria and culture conditions

*S. mutans* ATCC25175 and *S. sobrinus* ATCC33478 were maintained as frozen stock cultures and were cultured anaerobically at 37 °C in a solid trypticase soy broth (BBL, Cockeysville, MD, USA), yeast extract (Difco Laboratories, Detroit, MI, USA), and agar for 18 h. Afterward, the microorganisms were inoculated in 150-ml liquid TSBY for 18 h and were then anaerobically inoculated separately from the 150 ml of liquid TSBY with a radioactive marker, 74 kBq of [6-^3^H] thymidine, used to codify the microorganism and cultured for 18 h at 37 °C. Next, bacteria were collected through centrifugation of 8000×g for 15 min at 4 °C into 0.05 M phosphate-buffered saline (PBS) adjusted to pH 7.0 and washed three times with PBS. The concentration of *S. mutans* and *S. sobrinus* was 105 CFU/ml.

### Sample analysis

The orthodontic brackets were dispersed from the cap of a glass mold and immersed in 150 ml of *S. mutans* (150 brackets) and *S. sobrinus* (150 brackets) radiolabeled fluid, respectively, at 37 °C for 2 h in constant movement. To remove the non-adhering bacteria, the brackets were removed from the glass mold and washed three times with PBS.

The labeled bacteria that adhered to the brackets were collected using an automatic sample combustion equipment (ACS-113, Aloka, Tokyo, Japan). Tritium was recovered as H_2_O in Aquasol-2 (Packard), and radioactivity was measured using a liquid scintillation counter (LSC-900, Aloka) [33–35]. The results were recorded as disintegration per minute (dpm); therefore, the average of higher radiation level was proportional to the higher level of bacterial colonization.

In addition, after submerging the specimens for 2 h at 37 °C in a solution containing cultured microorganisms with continuous stirring, some representative samples were observed under a scanning electron microscope (SEM) at ×2500 and ×5000 magnifications for qualitative analysis. For the SEM observation, the samples were chemically prefixed with glutaraldehyde and fixed with osmium tetroxide, dehydrated with an ascending series of ethanol, and freeze-dried. The samples were coated with a thin layer of osmium [20, 24, 36].

### Statistical analysis

The data were registered and examined using a software for statistical analyses (SPSS 21, International Business Machines Corp, NY, USA). The differences in the measured values among the orthodontic brackets were tested by one-way analysis of variance (ANOVA) with a Scheffé test for multiple comparisons. A probability of less than 0.05 for similarity of distribution was considered to be statistically significant.

## Results

### Adhesion of *Streptococcus mutans*

The adherence of *S. mutans* radiolabeled to orthodontic brackets were significantly different between the groups (*p* ≤ 0.05). The scores were expressed as dpm as shown in Table 1. The dpm values, orthodontic brackets with greater adhesion of *S. mutans* were labeled as group GVI (3153.83 dpm), followed by group GVIII (2203.94 dpm), and finally group GIX (2186.23 dpm), silver nanoparticles are not added to these groups. Moreover, the groups with the lowest bacterial adherence were those with a coaggregation of silver nanoparticles; these groups are as follows: group GVn (687.33 dpm), followed by group GIIn (599.13 dpm), and group GIIIn (563.01 dpm).

**Table 1:** Quantitative test to *S. mutans* by radiolabeled (^3^H)

Bracket	DPM^a^	SD^b^	Sheffè test^c^
GIn In Vu Ag	707.78	(265.29)	A
GIIn Alexander Ag	599.13	(260.85)	A
GIIIn Gemini Ag	563.01	(287.71)	B
GIVn Nu-Edge Ag	775.39	(520.47)	B
GVn Radiance Ag	687.33	(284.24)	B
GVI In Vu	3153.83	(1071.06)	C
GVII Alexander	2044.00	(904.52)	D
GVIII Gemini	2203.94	(868.32)	C
GIX Nu-Edge	2186.23	(568.11)	C
GX Radiance	1714.01	(375.42)	C, D

^a^DPM (disintegration per minute)

^b^SD (standard deviation)

^c^Orthodontic brackets with different letters are significantly different from each other

### Adhesion of *Streptococcus sobrinus*

The adherence of *S. sobrinus* radiolabeled to orthodontic brackets were significantly different between the groups (*p* ≤ 0.05). The scores were expressed as dpm as shown in Table 2. For the dpm values, the orthodontic brackets with greater adhesion of *S. sobrinus* were grouped as follows: group GIX (8197.32 dpm), group GVIII (7518.39 dpm), and group GVI (7256.29 dpm), silver nanoparticles are not added to these groups. Moreover, the groups with the lowest bacterial adherence were those with a coaggregation of silver nanoparticles; the groups were as follows: group GVn (1085.67 dpm), group GIVn (1084.31 dpm), and group GIIIn (1044.08 dpm).

**Table 2:** Quantitative test to *S. sobrinus* by radiolabeled (^3^H)

Bracket	DPM^a^	SD^b^	Sheffè test^c^
GIn In Vu Ag	1906.48	(1037.91)	A
GIIn Alexander Ag	1513.64	(882.12)	A
GIIIn Gemini Ag	1044.08	(415.86)	B, C
GIVn Nu-Edge Ag	1084.31	(415.44)	B, C
GVn Radiance Ag	1085.67	(303.03)	B, C
GVI In Vu	7256.29	(1421.48)	D
GVII Alexander	5457.09	(1550.05)	E
GVIII Gemini	7518.39	(1494.52)	D
GIX Nu-Edge	8197.32	(2174.98)	D
GX Radiance	6660.28	(1436.74)	D,E

^a^DPM (disintegration per minute)

^b^SD (standard deviation)

^c^Orthodontic brackets with different letters are significantly different from each other

**Fig. 1:**
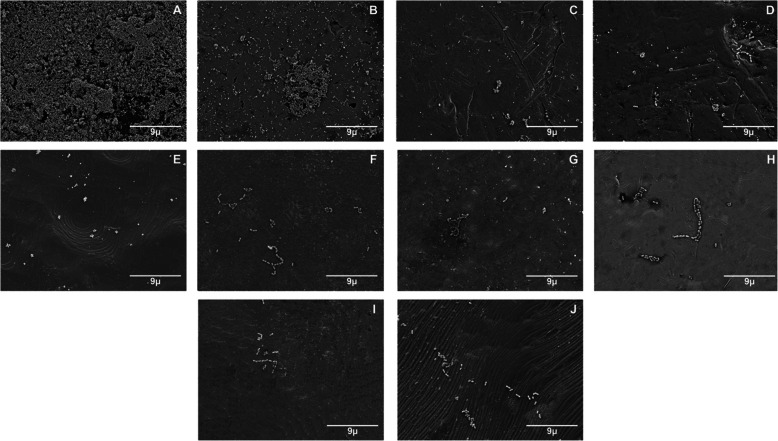
Representative images from SEM of orthodontic brackets exposed to *S.mutans* (×2500). **a** GIn. **b** GIIn. **c** GIIIn. **d** GIVn. **e** GVn. **f** GVI. **g** GVII. **h** GVIII. **i** GIX. **j** GX

**Fig. 2:**
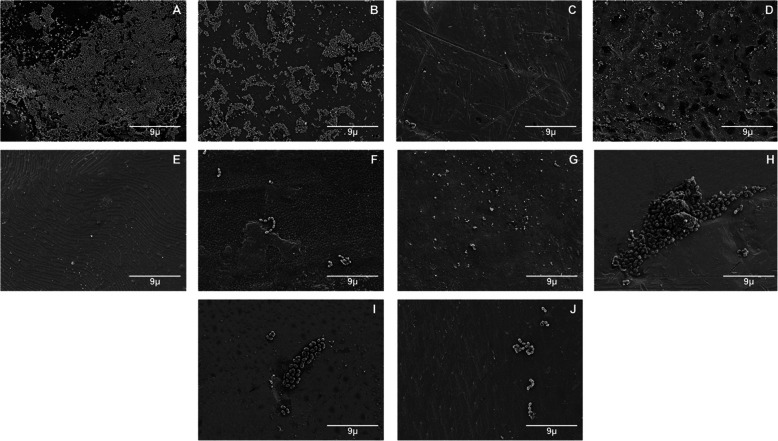
Representative images from SEM of orthodontic brackets exposed to *S.sobrinus* (×2500). **a** GIn. **b** GIIn. **c** GIIIn. **d** GIVn. **e** GVn. **f** GVI. **g** GVII. **h** GVIII. **i** GIX. **j** GX

The representative SEM images of the brackets materials obtained after 2 h of immersion in *S. mutans* and *S. sobrinus* solutions are shown in Figs. 1 and 2. The results obtained in the quantitative analysis are consistent with the qualitative observation in SEM.

According to the dpm values, for all the bracket groups with added silver nanoparticles (AgNPs), the bacterial adherence of both microorganisms was lower than that in the groups of brackets without the addition of silver nanoparticles.

## Discussion

White spot lesions are associated with enamel demineralization around fixed orthodontic appliances. Orthodontic appliances have a leading role in the demineralization of enamel because they provide greater retention of biofilm, providing more surfaces for bacterial adhesion, and its complex design prevents adequate tooth surface during cleaning [20, 32, 36]. Several species of bacteria are involved in the formation of dental biofilm, and white spots lesions caused by organic acids that secrete cariogenic bacteria. Among them, *S. mutans* and *S. sobrinus* have been recognized as the prime causative organisms of dental caries [2]. Gorelick reported that enamel demineralization occurs from the first month after the placement of fixed appliances, and it is estimated that the prevalence of white spot injury in the enamel of orthodontically treated patients ranges from 12.6 to 50% [16, 17].

Limited information is available on which orthodontic bracket are most susceptible to adhesion of cariogenic streptococcus. *Streptococcus mutans* and *Streptococcus sobrinus* are mainly responsible for dental caries. Because the popularity of plastic brackets has grown during the last few years due to increased demand for superior esthetics during orthodontic treatment, the purpose of this study was to identify possible variations in the adhesion patterns of *S. mutans* and *S. sobrinus* on different bracket materials to decrease the risk of possible side effects, such as the development of white spot lesions.

The results showed a significant difference in the level of adhesion between both bacterial species (Tables 1 and 2). In general, adhesion to the materials tested was greater for *Streptococcus sobrinus* than for *Streptococcus mutans.* This differs in a previous study, which reported that *S. mutans* have more adherence to orthodontic brackets than *S. sobrinus* and that each species of cariogenic streptococci has a characteristic level of adhesion [37].

Velázquez et al. reported in their study the bacterial adhesion to different types of orthodontic composites, these resins retain biofilm, and the finding that the Blugloo resin of the Ormco brand obtained the highest level of bacterial adhesion and that *S. mutans* and *S. sobrinus* can generate the higher risk of white spot injury [18].

The bacterial adhesion of *S. mutans* and *S. sobrinus* to orthodontic attachments is caused by Van der Waals forces, electrostatic and hydrophobic interaction, also it has been reported that the adhesion of cariogenic streptococcus to orthodontic attachments, such as orthodontic composites, elastomeric chains, and brackets, is caused by their manufacturing materials and their complex design. These can retain more amount of biofilm that is highly colonized by *S. mutans* and *S. sobrinus* around the fixed appliances and can proliferate on tooth surfaces and develop dental caries. This study was set to accurately determine the level of *S. mutans* and *S. sobrinus* that adhere to the orthodontic brackets. When cultured and tested independently, it is seen that both microorganisms are directly related to dental caries and also are the biggest acid producer, which causes demineralization [36].

In recent studies, surface modification of stainless steel orthodontic and NiTi alloy wires with AgNPs has led to antibacterial positive results against Lactobacillus acidophilus: an in vitro study [12].

Moreover, Eliades et al. [3, 19] identified stainless steel as a surface material with an increased potential for microbial attachment after measuring the free surface energy and the work of adhesion of raw materials and compared it with polycarbonate and ceramic materials. In contrast, results from Fournier et al. [3, 20] indicate weaker in vitro affinity of *S. mutans* for metallic brackets than for plastic brackets, which is in accordance with the results of a study conducted by Ahn et al. [2], who made multiple in vitro comparisons of cariogenic adhesion amounts on stainless steel, plastic, ceramic, and titanium brackets. Besides significant differences in the adhesion pattern of different cariogenic strains, their results showed higher adherence of cariogenic streptococci on plastic brackets than that in the four other types of brackets.

For this research, the samples were not coated with saliva because previous studies [2, 37] have described that saliva coating does not significantly modify the adhesion of *S. mutans* and *S. sobrinus*. This report is similar to other investigations, which show that the saliva coating does not significantly alter the adhesion of *Streptococcus* in the underlying materials [36, 38].

Orthodontics is one of the treatments most requested by patients; however, as mentioned above, because of the complexity of its attachments, it generates more bacterial colonization and development of white spot lesions. Fluor has been used as a preventive method, but it has not been enough to avoid its occurrence [39, 40]. For this reason, at present, it is necessary to incorporate antibacterial substances, such as silver, making use of nanotechnology. Nanotechnology has been widely used for biomedical purposes, ranging from diagnosis, treatment, medication administration, to the coating of medical devices and personal health care. With the increased application of NPs in the medical context, it is necessary to have a better understanding of the mechanisms of NPs biological interactions and their potential toxicity, as well as the unique physiochemical properties of NPs, such as antibacterial, antifungal, antiviral, and anti-inflammatory activity [14].

The nanoparticles of metals, such as silver, copper, gold, titanium, and zinc, have gained significant interest in the recent years because of their remarkable antibacterial properties and because each has different properties and activation spectrum. For many years, silver has been employed as a bacteriostatic agent, so it was found to be a versatile application in the care of human health.

Silver nanoparticles are nanostructured materials whose base is silver salts. It has different biomedical applications because of its high antibacterial effect, aside from producing null toxicity in human tissues when used in low concentrations, which is why it is widely used in medical areas, such as covering materials, wound dressings, bone cement, food supplements, catheters, and in dentistry, they are used in some dental materials, such as pastes, cement, adhesives, resins, and dental implants [14].

The antibacterial effect of these nanostructured agents is attributed to the high surface area of the nanoparticles, which allows the greater presence of atoms on the surface, providing maximum contact with the environment [41]. Furthermore, Garcia and colleagues report in their study that the small size of these particles makes penetration through cell membranes easier (inhibiting ADN synthesis) [38]. Studies show that the positive charges of metal ions are critical for the antibacterial activity, allowing electrostatic action between the negative charge of the cell membrane of the bacteria and the positive charge of the nanoparticles [14, 41].

Different synthetic AgNPs routes lead to variable sizes, shapes, morphology, and even stability. Generally, these methods can be classified into three broad categories: physical, chemical, and biological (or green) syntheses.

The chemical method used in this research was suggested by Tanusheree Bala in their report [42]. Also, the use of equipment and methodology, such as the automatic sample combustion machine and the liquid scintillation counter device for measuring ^3^H, which are amply described by Saku et al. [33], and Nagayama et al. [34], as well as the results expressed and recorded in dpm. In this sense, a higher value of dpm means higher radioactivity, and therefore, higher adherence of a radiolabeled microorganism is found. In contrast, lower values of dpm indicate lesser adherence of the radiolabeled microorganism.

The results (Tables 1 and 2) in this study showed that the adherence of *S. mutans* and *S. sobrinus* radiolabeled to orthodontic brackets were significantly different between groups for both microorganisms (*p* ≤ 0.05). In general, the cariogenic streptococcus adhered to the orthodontic brackets with silver nanoparticles significantly less than to the bracket without silver nanoparticles. Group GIIIn (563.01 dpm) for *S. mutans* and group GIIIn (1044.08 dpm) for *S. sobrinus* have the lowest bacterial adherence for both microorganisms. In the same mode, group GVI (3153.83 dpm) and group GIX (8197.32 dpm) had the highest bacterial adherence. In general, the level of bacterial adhesion to the materials tested was greater for *S. sobrinus* than that for *S. mutans*.

It is also important to remark that group GIIIn (Gemini Roth) showed the lowest bacterial adherence for both microorganisms, it is suggested that this may be caused by several factors. First, this group, in specific, as it can be observed in the images of the SEM, presents a smoother surface, with a better finish, the rough surface increases the surface area and niches, which are suitable environments for bacterial adhesión [43]. In addition, the literature reports that the positive charges of the metal ions repel the negative charges of the bacterial membrane. This could be due to the highest coaggregation of AgNPs that is why it has the highest antibacterial potential, and its significant reduction of microorganism adhesion has become an excellent option for orthodontic treatments with a wide possibility of avoiding dental caries and also the development of white spot lesion.

## Conclusions

1. The silver coating decreased the adhesion of both *S. mutans* and *S. Sobrinus* to the orthodontic brackets, which demonstrates their antibacterial properties.

2. The modification of the surface of orthodontic brackets with silver nanoparticles can modify to prevent the development of dental plaque and dental caries during orthodontic treatment.

## Data Availability

The datasets used and/or analyzed during the current study are available from the corresponding author on reasonable request. All data generated or analyzed during this study are included in this published article (and its supplementary information files).

## Abbreviations

WHO: World Health Organization
AgNPs: Silver nanoparticles
*S.* mutans: Streptococcus *mutans*
*S. sobrinus*: *Streptococcus sobrinus*
ANOVA: Analysis of variance
NPs: Nanoparticles
